# A systematised review and evidence synthesis on the broader societal impact of vaccines against *Salmonella*

**DOI:** 10.1038/s41541-024-01034-4

**Published:** 2025-02-01

**Authors:** Ezgi Dilek Demirtas, Rosanna Barnard, Jungseok Lee, Mark Jit

**Affiliations:** 1 Department of Infectious Disease Epidemiology, Faculty of Epidemiology and Population Health, London School of Hygiene & Tropical Medicine, London, UK; 2 International Vaccine Institute, Seoul, South Korea; 3 Department of Global and Environmental Health, School of Global Public Health, New York University, New York, United States of America

**Keywords:** Bacterial infection, Outcomes research, Epidemiology

## Abstract

Vaccines against *Salmonella* Typhi are available, while vaccines against invasive non-typhoidal *Salmonella* are in development. Investments in vaccine development and introduction need to be informed by a full value of vaccines assessment, including consideration of broader societal impacts of salmonellae disease. We reviewed literature on these broader impacts in low- and middle-income countries to inform a conceptual framework. We found 16 studies relevant to *Salmonella*, but only one study on non-typhoidal *Salmonella*. Despite variations in study design, methodology, and study quality, salmonellae infections were largely associated with negative broader societal impacts, including detriments in childhood physical development (very weak association), childhood educational development (strong to very strong association), household security (moderate association), public health spending (moderate association), and national income (moderate to strong association). Study quality was low for all impacts except childhood physical development. There were no studies measuring economic impact of antimicrobial resistance, changes in household behaviour or health inequalities.

## Introduction

The bacteria species *Salmonella* is classified into two groups: typhoidal (consisting of serotypes Typhi and Paratyphi A, B and C) and non-typhoidal. Typhoidal *Salmonella* and some non-typhoidal *Salmonella* serotypes can cause invasive disease. In high-income settings, non-typhoidal *Salmonella* usually causes a self-limiting diarrhoeal disease in healthy individuals^1^. By contrast, in sub-Saharan Africa, due to a high prevalence of comorbidities within the population such as malnutrition, HIV, sickle cell disease and malaria, invasive non-typhoidal *Salmonella* (iNTS) with a high case-fatality risk is common^1,2^. Often, iNTS presents as fever alone making it difficult to diagnose without blood culture, which is a limited resource in low- and middle-income countries (LMICs). The 2017 Global Burden of Disease study estimated 535,000 iNTS cases and 77,500 deaths, with the majority of cases believed to occur in sub-Saharan Africa, followed by south Asia. Antibiotic resistance in iNTS is a growing problem, with multidrug resistance common in sub-Saharan Africa and sometimes accounting for the majority of detected cases^3^.

Several vaccines against *Salmonella* Typhi are available and are recommended in disease endemic areas. While a 2020 global market study for typhoid vaccines conducted by the World Health Organization (WHO) estimated a market for typhoid conjugate vaccine (TCV) in up to 94 countries^4^, to date, TCV has only been introduced nationally into the routine immunisation schedule in 5 countries^5^. Currently, there is no vaccine available for non-typhoidal *Salmonella*. However, several polyvalent Typhi/NTS vaccines are in development^6^, including a trivalent vaccine against *Salmonella* Typhi and two of the most invasive non-typhoidal serotypes (Typhimurium and Enteritidis).

Health economic considerations such as cost-effectiveness are key pieces of evidence to inform new vaccine adoption. However, most economic evaluations of vaccines only focus on direct health and short-term economic benefits on vaccinated individuals and closely related individuals such as caregivers. Vaccines may have broader societal benefits, such as protecting non-vaccinated individuals through herd effects, reducing antibiotic resistance rates, improving child development and educational outcomes, enhancing family planning, and strengthening macroeconomic stability. Several conceptual frameworks have been proposed to categorise these broader societal benefits, both for vaccines in general^7,8^ and for specific vaccines such as those against Group A *Streptococcus*, *Haemophilus influenzae* and *Neisseria meningitidis*^9–11^. These broader societal effects are captured in the 2023 Full Value of Vaccine Assessment framework developed by WHO to inform investment in vaccine development and introduction, particularly in resource-constrained settings^12^.

The aim of this paper is to review published literature on the broader societal impacts of salmonellae infections, including the broader societal impacts of antimicrobial resistance where reported. These studies are then used to define pathways between Salmonellae infections and broader societal impacts that could be addressed by vaccines, assess the strength of evidence behind each pathway, and use this information to develop a conceptual framework for the broader societal benefits of *Salmonella* vaccines, as an essential step towards a full value of vaccines assessment.

## Results

**Fig. 1:**
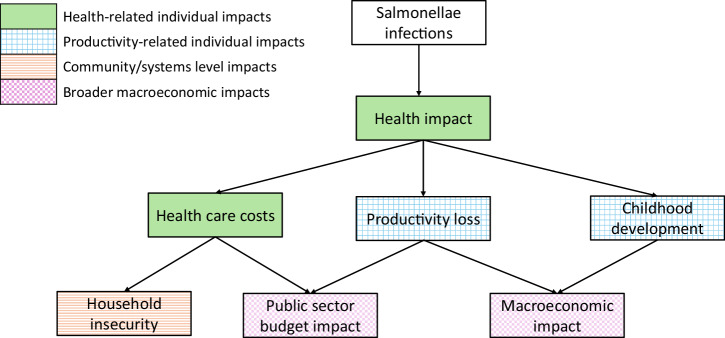
Conceptual framework of the pathways to the broader economic impacts of *Salmonella* disease. Adapted from 2011 framework^7^, but with pathways that do not have evidence from *Salmonella* literature removed.

We identified 1812 records from our database review, but only 16 studies met our inclusion criteria (see Fig. 1 for the PRISMA flow diagram). Of the 91 articles double reviewed by the senior author, 91/92 (98.9%) reached the same conclusion about inclusion. For the single article with differing conclusions, the senior author aligned with the reviewer’s conclusion after discussion. Of the 24 fields (12 fields across 2 articles) double extracted by the senior author, 23/24 (95.8%) had consistent conclusion, albeit with variations in phrasing. In the single field with divergent conclusions, the reviewer concurred with the senior author’s conclusion after discussion.

We found studies in 21 Asian countries, 11 African countries, and 1 South American country. This corresponded to 21 lower-middle, 8 upper-middle, and 4 low-income countries as classified by the World Bank in the year of the study period. Three studies estimated economic gains in multiple LMICs. Data were collected in various time periods between 1980 and 2020. Half of the studies included several salmonellae serotypes. 15 studies collected data on *S.* Typhi, 8 on *S.* Paratyphi, and 1 on NTS. There were 7 ecological studies, 4 case-series, 3 cross-sectional surveys, 1 cohort study and 1 case-control study. The most commonly identified broader societal impact was household security, followed by childhood development, macro-economic impacts, public sector budget impacts, and productivity-related impacts (see Table 1). No studies examined community impacts, social impacts, equity, or economic impact of antimicrobial resistance. Most (13/16) studies were published in or after 2018. Only a few studies reported statistical measures of association (see Supplementary Table 9).

**Table 1:** Framework for the broader societal impacts of *Salmonella* disease, with the number of articles identified in each impact category

Category	Definition	Number of articles
A. Health-related benefits to vaccinated individuals		
A1. Health gains	Reduction in morbidity and mortality	Not in scope
A2. Health care cost savings	Reduction in direct cost of health care borne by the public sector or private individuals	Not in scope
B. Productivity-related benefits		
B1. Productivity gains related to care	Reduction in lost days of work due to sickness or caring for a sick patient	1
B2. Productivity gains related to health effects	Reduction in lost days of work due to sickness or death of sick patient	1
B3. Productivity gains related to non-utility capabilities	Increased lifetime productivity because of enhanced capabilities (such as improved cognition and educational attainment) not easily measured using utility-based preference measures	11
C. Community or health systems externalities		
C1. Ecological effects	Health improvements in unvaccinated community members as a result of ecological effects such as herd immunity, eradication and reduced antibiotic usage.	0
C2. Equity	More equal distribution of health outcomes	0
C3. Financial and programmatic synergies and sustainability	Improved financial sustainability as a result of effects such as synergies with other health care programmes (eg. delivery platforms), stimulation of private demand and mechanisms to enhance group purchasing power (eg. PAHO revolving fund)	0
C4. Household security	Improved financial security of household as a result of reduced risk of catastrophic expenditure	14
D. Broader economic indicators		
D1. Changes to household behaviour	Economic improvements due to changes in household choices such as fertility and consumption/saving as a result of improved child health and survival	0
D2. Public sector budget impact	Change to an individual’s net transfers to the national budget over his/her lifetime.	2
D3. Short-term macroeconomic impact	Changes to national income or production as a result of short-term exogeneous shocks to the economy.	5
D4. Long-term macroeconomic impact	Changes to national income or production as a result of long-term changes to drivers such as labour supply and foreign direct investment	

Some articles appear in multiple categories, so the column total for "Number of articles" adds to more than the total number of articles identified in the review (i.e. 16). Category labels (e.g. “B1”) correspond to labels used in the 2011 framework^7^.

Table 2 summarises the risk of bias for each included study according to ROBINS-E. Study quality grades and their assessment process according to the quality checklists listed in the Methods are shown in Supplementary Note 1 and Supplementary Tables 4–6. Among 17 quality appraisal assessments (one study reporting on both quantitative and qualitative data was assessed by two checklists), 1 prospective case-control study was graded as very good quality, 4 studies (1 cross-sectional survey and 3 case-series) as good quality, and 12 (7 ecological studies, 2 cross-sectional surveys, and 3 case-series) as low quality. Supplementary Table 7 summarises this review’s findings per impact category, by describing which study identified which societal impacts, the strength of associations reported, and the overall assessment of study. Supplementary Table 8 shows the time at which infection was recorded, and childhood physical or educational development was measured.

**Table 2:** Risk of bias for each study, as assessed by the ROBINS-E tool

	D1	D2	D3	D4	D5	D6	D7	Overall
Akinyemi et al.^16^	Very high	Low	High	Low	Low	Low	Low	Very high
Balaji et al.^17^	Very high	Low	High	Low	Low	Low	Low	Very high
Bhutta et al.^18^	Very high	Low	High	Low	Low	Low	Low	Very high
Das et al.^15^	Very high	Low	High	Low	Low	Low	Low	Very high
Das et al.^14^	Low	Low	Some concerns	Low	Low	Low	Low	Some concerns
Kaljee et al.^13^	High	High	Low	Low	High	Some concerns	Low	Very high
Keddy et al.^19^	Very high	Low	High	Low	Low	Low	Low	Very high
Kumar et al.^24^	Low	High	High	Low	Low	Some concerns	Low	High
Limani et al.^28^	Very high	Low	High	Low	Some concerns	High	Low	Very high
Onuche et al.^30^	Very high	Some concerns	Some concerns	Low	Low	High	Low	Very high
Poulos et al.^22^	High	Low	High	Low	Very high	High	Low	Very high
Rahman et al.^23^	Very high	Some concerns	High	Low	Low	Some concerns	Low	Very high
Rahman et al.^27^	Low	Some concerns	Some concerns	Low	Low	Some concerns	Low	Some concerns
Saha et al.^20^	Very high	Low	High	Low	Low	Low	Low	Very high
Seyi-Olajide et al.^29^	High	Low	High	Low	Some concerns	Low	Low	Very high
Techasaensiri et al.^21^	Very high	Low	Some concerns	Low	Low	Low	Low	Very high

D1: Bias due to confounding

D2: Bias arising from the measurement of the exposure

D3: Bias in selection of participants into the study (or into the analysis)

D4: Bias due to post-exposure interventions

D5: Bias due to missing data

D6: Bias arising from measurement of the outcome

D7: Bias in selection of the reported result

### Productivity-related impacts related to care and health effects (B1 and B2) (1 study)

#### Evidence that salmonellae infections negatively impact non-market productive activities (no association explored, quality of evidence low to moderate)

A case-series in Nepal^13^ of respondents who were patients or caregivers of patients with blood-culture-confirmed typhoid in the past six months found that 70% of respondents reported a mean loss of 12 (range 1–28) days performing routine household tasks. Respondents also reported in qualitative interviews that patients may lose educational or work opportunities, and that care of patients required extensive time from household members and their social network. Limitations were that the authors did not specify if these respondents were patients or caregivers, and there was no control group (so statistical association could not be explored).

### Childhood physical development (B3) (1 study)

#### Evidence that salmonellae infections impact childhood physical development (strength of association very weak, quality of evidence high)

A prospective case-control study in seven Asian and African countries^14^ compared children under five years with non-typhoidal *Salmonella* detected in their stool, with uninfected sex- and age-matched controls from the same or neighbouring communities in 2007–2011. It found that having non-typhoidal *Salmonella* was associated (but only very weakly) with having low weight-for-age, weight-for-height and height-for-age. The study was assessed as being of high quality as it included controls, culture-confirmed cases, a large sample size, randomly selected participants, and repeated measurements to exclude reverse causality. A limitation was potential confounding due to lack of data to adjust for maternal body mass index, gestational age, birth weight, serum nutrient level, and child HIV status.

### Childhood educational development (B3) (9 studies)

#### Evidence that salmonellae infections impact childhood educational development (strength of association very strong, quality of evidence low)

An ecological study in Pakistan^15^ found that national adult female literacy and adult literacy were very strongly associated with decreases in blood-culture-positive typhoid cases from a selection of laboratories. However, this was the only one of the 9 studies in this category to assess the strength of association. Seven ecological studies^14,16–21^ reported increasing adult female literacy over time, while typhoid and paratyphoid isolate trends varied from decreasing to increasing. A case series in five Asian countries in 2001–2004^22^ found that 31–76% of children with blood-culture-confirmed typhoid missed days of school. A case-series in Nepal in 2015^13^ found that 45% of children with blood-culture-confirmed typhoid missed days of school, and that female community health volunteers at a rural public health centre reported educational development impacts on caregivers.

### Household insecurity (C4) (14 studies)

#### Evidence that salmonellae infections negatively impact household security (strength of association moderate for distress financing, conflicting results for poverty, not examined for catastrophic health expenditure and food insecurity, quality of evidence low)

Distress financing (defined as financing by borrowing money with or without interest, ex-gratia payments by relatives, selling assets, taking on additional paid work, reducing expenditure on food or removal of children from school) was examined in three studies. A cross-sectional study in Bangladesh^23^ found that 25.7% of households self-reporting typhoid in the previous 30 days resorted to distress financing, giving an adjusted relative risk of distress financing of 1.92 (95% CI 1.08–3.43). A case-series in India^24^ found that 45% of patients hospitalised with blood-culture confirmed typhoid or paratyphoid, and 66.7% of patients with confirmed enteric fever perforations, resorted to distress financing. A case-series in Nepal^13^ found that 15% of households that experienced blood-culture-confirmed typhoid cases reported distress financing.

Extreme poverty (defined by the World Bank as living on less than 1.90 USD/day in 2011 USD^25^) was analysed in six ecological studies assessed as being of low quality. An ecological study in Pakistan in 1990–2015^15^ found that poverty was strongly associated with blood-culture-confirmed typhoid incidence but poverty was not associated with paratyphoid incidence. An ecological study in Thailand in 2003–2014^21^ found no evidence of a significant association between poverty and culture-confirmed typhoid and paratyphoid cases collected through the national enteric fever surveillance programme (including urine, stool and blood samples). Five other ecological studies^14,16,18–20^ found that poverty decreased over time while typhoid and paratyphoid incidence ranged from decreasing to increasing over time.

Catastrophic health expenditure (CHE, defined by WHO as out-of-pocket payments greater than 40% of capacity to pay for healthcare^26^) was explored in three studies without controls. A cross-sectional study in Bangladesh in 2011^27^, which was assessed as being of high quality, estimated that 24.1% (95% credible interval 10.5–40.7) of typhoid patients experienced CHE after adjusting for confounders, which was the highest estimated proportion among over 35 illnesses recorded. A low-quality case-series in Malawi^28^ estimated that 44% of households with blood-culture-confirmed typhoid requiring hospitalisation suffered CHE (due to non-medical and indirect costs) despite the free government-provided healthcare. A low-quality case series in Nigeria^29^ reported that all families of 32 child patients receiving surgery for typhoid perforation experienced CHE. In addition to the three studies directly examining CHE, a cross-sectional study in five Asian countries found that privately-borne costs for hospitalised cases could be up to 15% of annual household income^22^.

A cross-sectional survey of rural households in Nigeria^30^, assessed as being of low quality, examined food security (which the study defined as the availability, accessibility, affordability, and sustainability of food in the quality and quantity required by people). This study found that typhoid experienced by the household head during the farming season accounted for the highest food insecurity headcount (73.3%) and severity (34.3%) among several diseases. Study limitations include not adjusting for confounding, not stating the sample size and not giving information on how typhoid was diagnosed.

### Public sector budget impact (D2) (2 studies)

#### Evidence that salmonellae infections impact public health spending (strength of association moderate for *S.* Typhi, no evidence of association for *S.* Paratyphi, quality of evidence low)

Two studies examined the association between *Salmonella* infections and health expenditure on a national level (i.e. not simply health expenses directly related to treating the disease episode).

An ecological study in Pakistan^15^ found that annual national health expenditure as a proportion of gross domestic product (GDP) was moderately associated with decreases in blood-culture-positive typhoid cases from a selection of laboratories, but not with blood-culture-positive paratyphoid cases (*r* = 0.09, *p* = 0.70). Strengths of the study were a large sample size (*n* = 17387 for typhoid, 8286 for paratyphoid), laboratory confirmation, and long time period (1990–2015). Limitations include the ecological nature of the study (with the whole country being the unit of analysis for health expenditure), selection bias due to exclusion of cases not leading to positive blood culture, and lack of adjustment for confounding.

An ecological study in India^17^ found that public spending on health increased in 1995–2010 while blood-culture-confirmed typhoid and paratyphoid cases from three tertiary hospitals decreased over the same time period. No statistical associations were explored, nor were confounding or selection biases examined.

### Macro-economic impacts (D3 and D4) (4 studies)

#### Evidence that salmonellae infections impact macro-economic indicators (strength of association moderate to strong, quality of evidence low)

An ecological study in Pakistan^15^ found that annual gross national income (GNI) per capita was associated with decreases in blood-culture-positive typhoid and paratyphoid cases from a selection of laboratories. Ecological studies in India^17^, Nigeria^16^ and South Africa^19^ showed simultaneous increasing GNI or GDP and decreasing typhoid and paratyphoid incidence, without measuring the strength of association. None of these studies assessed the direction of causality or considered selection biases or confounding. However, the Nigerian study^16^ noted gaps in medical records due to strikes and irregular record keeping.

## Discussion

To our knowledge, this is the first systematised review to investigate and categorise the broader societal impacts of salmonellae infections. It found that salmonellae infections were associated with adverse impacts on productivity, childhood education and physical development, household financial security and national income. However, our conclusion is constrained by limitations within the existing body of literature, including the scarcity of relevant studies, methodological heterogeneity (in study design, exposure measurement, and outcome measurement), confounding and unclear direction of causality. Most studies were not able to assess the strength of associations because of lack of control groups or absence of any statistical analyses. We assessed the quality of evidence as generally low and at best moderate, apart from one study that found an association between non-typhoidal *Salmonella* infections and childhood physical development^14^. That one high quality study highlights many of the characteristics that could be included in future studies to improve their quality, including sex- and age-matched controls, culture-confirmed cases and repeated measurements over time to exclude reverse causality.

The broader societal impacts of *Salmonellae* infections explored by the studies we identified in this review were almost all detrimental. Incorporating these impacts into cost-effectiveness analyses and/or into a full value of vaccines assessment would therefore increase the value to stakeholders and health decision makers of a vaccine that reduced salmonellae infections. The only broader societal impact of salmonellae infections which was not detrimental was on public sector budgets. In the studies included in our review, decreasing typhoid incidence was associated with increasing health expenditure. However, this evidence was assessed to be of low quality. Furthermore, for all of these studies, data collection on the two variables of interest (typhoid incidence and health expenditure) was conducted in the same time period, so the relationship between *Salmonella* burden and public sector budgets may have been driven by reverse causality i.e. spending more on health reduces salmonellae infections.

Many pathways within the broader societal impact framework have plausible mechanisms. *Salmonella* infections lead to health conditions (although we do not directly investigate this relationship). Such conditions necessitate treatment and hinder patients’ ability to work. This can increase national healthcare expenditures if costs are covered by the public sector, or jeopardise household financial security if the burden falls on patients/caregivers. Conversely, increased national healthcare spending may facilitate timely treatment for patients, thereby reducing the risk of onward transmission. Diarrheal infections have been associated with malnutrition^31^ and school absenteeism^13,22^, which can both adversely affect physical and educational development.

Our screening process found that broader societal impacts are rarely incorporated into studies on salmonellae infections, despite our broad inclusion criteria which ensured that a variety of study designs and measurement techniques were included. More than half of the 1812 studies returned in the initial search were excluded because they did not report on any broader societal impacts. Indeed, we did not identify any studies assessing the impact of salmonellae infections on many of the impact categories outlined in previous frameworks^7,8^, such as economic impact of antimicrobial resistance, changes in household behaviour, and health inequalities. However, the absence of evidence to support many of these causal pathways identified for other vaccine-preventable diseases does not imply evidence for the absence of such relationships, since we did not find any negative studies (i.e. studies that tried and failed to find such a relationship) either.

Only one of 16 studies identified by our review considered the impacts of non-typhoidal *Salmonella*, which emphasises the need for additional evidence to inform the next generation of polyvalent vaccines, particularly those targeting invasive non-typhoidal *Salmonella*. Broader societal impacts have been highlighted as important aspects to inform decision-making around vaccine development and introduction^12^, suggesting that more high-quality research is needed to explore the broader societal impacts of salmonellae infection and the potential benefits of vaccines. Pathogen surveillance systems could provide insight on how societal outcomes of *Salmonella* change over time, which could help track the long-term impacts of vaccines as they are introduced.

There may be several reasons for the lack of evidence on the broader societal impact of salmonellae infections. First, vaccine trials focus on immediate clinical outcomes, and do not routinely incorporate cognitive, educational, and behavioural endpoints. This may reflect both lack of awareness of the importance of assessing such outcomes to vaccine investment decisions and the long follow-up period that would be needed in order to measure the impact of vaccines on some of these outcomes (such as school results or earnings in adulthood). Hence, the existing body of evidence consists largely of retrospective observational cohorts which are subject to bias and reverse causality, cost-of-illness studies with limited use for causal inference, theoretical models, and conjectural discussions. Second, the review itself had several limitations related to its systematised but not systematic nature. Screening and data extraction were conducted by a single reviewer because of resource and time constraints, although 5% of articles for screening and for data extraction were checked by a second reviewer who found minimal discrepancies. Still, study classification and quality assessment may have been misjudged, as these are inherently subjective processes. To make the search feasible, we used relatively focused search terms related to previously identified categories of broader societal impact, rather than searching the entirety of the socioeconomic literature on *Salmonella* disease.

The scarcity of existing evidence on broader societal impacts may hinder the completeness of the full value estimation of vaccines. This suggests that the benefit of (and methodology for) exploring broader vaccine impacts needs to be made clearer in comprehensive guidelines for vaccine evaluations, to encourage high-quality research in this area. If guidelines were available on how to incorporate such outcomes into vaccine evaluations (including economic evaluations such as cost-effectiveness and cost-benefit studies), this would encourage primary research to generate the evidence to inform such analyses. Indeed, this knowledge gap can be easily addressed if additional tools to measure such outcomes were made available and more widely used. Shorter-term outcomes like household choices around consumption and savings, social impacts such as quality of life, and stigma could be routinely incorporated into clinical effectiveness trials of vaccines or into standard demographic and health surveys if suitable data collection instruments are developed. Studies collecting household data could also collect economic data by income or wealth quintile to enable analyses of the impact of vaccines on health equity.

In conclusion, despite important variations in study designs, methodology, strength of association, and study quality, salmonellae infections were largely associated with negative broader societal impacts. However, there is heterogeneity in the amount of evidence across different societal impacts, as well as differences in the number of studies which explore the impact of typhoidal and non-typhoidal *Salmonella*, which suggests that future research needs to prioritise generating evidence around the impact of salmonellae infections on less well-explored broader societal impacts, and particularly those impacts arising from invasive non-typhoidal *Salmonella* (see Table 3). The proposed broader societal impacts framework we have developed for *Salmonella* vaccines may contribute to better communication of the potential societal benefits surrounding future vaccine uptake, and offers valuable information to decision-makers, especially those outside of the traditional healthcare sector, such as ministries of education, finance, and national treasuries.

**Table 3:** Future research priorities around the broader societal impact of *Salmonella* vaccines

• Develop guidelines about how broader societal outcomes can be measured and incorporated into economic evaluations of *Salmonella* vaccines.
• Incorporate short-term broader societal outcomes (such as household choices, quality of life, stigma, and outcomes by socioeconomic quintile) into vaccine trials and household surveys.
• Develop study designs to collect longer-term broader societal outcomes (such as developmental outcomes) in ways that minimise bias.
• Develop surveillance systems to measure broader societal outcomes over time after vaccine introduction.

## Methods

This research was assessed by the LSHTM Research Governance & Integrity Office as not requiring ethical approval as it is a literature review involving only publicly available information.

### Overview

A mixed-methods systematised^32^ literature review was conducted. The Preferred Reporting Items for Systematic Reviews and Meta-Analyses (PRISMA) 2020 checklist^33^ was used to conduct and ensure clear reporting of the review (Supplementary Table 1).

### Search strategy

A single reviewer (the first author) searched Ovid MEDLINE and EconLit without time limitation, up to 12 April and 1 May 2023 respectively. The search strategy focused on two concepts: *Salmonella* disease, and its broader socio-economic burden. Each search concept used a combination of free-text terms (keywords and synonyms) and Medical Subject Headings (MeSH) to be as comprehensive as possible. Truncation, inclusion of words with alternative letters, absent letters, and proximity searching were applied when this would be likely to increase the sensitivity of the search. Subject headings were “exploded”, i.e. any more specific terms related to the subject heading that were relevant were also searched; otherwise, terms were selected individually. All subheadings were included. For example, the keyword search “(antibiotic resistance or antimicrobial resistance).mp.” and subject heading search “exp beta-lactam resistance/ or exp drug resistance, multiple, bacterial/ or exp trimethoprim resistance/” were used on MEDLINE to look for articles on antimicrobial resistance. Boolean operators AND/OR were used to combine results. Search filters for identifying papers specific to humans and to LMICs and written in the French and English languages were applied. Supplementary Tables 2–3 detail the search strategy.

### Selection criteria

We selected all English and French language studies set in LMICs that reported broader societal impacts of *Salmonella* infection in humans. We excluded studies that only reported health outcomes or traditional economic outcomes (direct medical or non-medical costs, productivity losses directly arising from illness episodes)^7^ and not wider societal impacts, as well as studies looking at antimicrobial resistance as an epidemiological outcome with no information on its societal impact. This is because most frameworks on broader societal impacts of vaccines^7,8^ consider health outcomes, direct health care costs, and productivity losses directly arising from illness episodes falling on patients and their household as “narrow” or “traditional” outcomes, since they are typically incorporated in standard economic evaluations. All empirical and modelling studies were included, but we excluded reviews, news articles, opinion pieces, commentaries, editorials, recommendations, methodology pieces and conference reports. We did not assess the direction of causality at the selection stage, i.e. studies reporting broader societal impact categories (e.g. macro-economic indicators, educational outcomes) were included whether they were considered as exposures for or outcomes of salmonellae infections by the study investigators.

### Data extraction

A standardised data extraction form was developed with the following categories: country/region, population studied, study period, *Salmonella* serotype(s), aim of study, study design and methods, identified impact(s) from the framework, and results. The strength of association, risk of bias and quality of each study was assessed. For two studies reporting unclear use of study design terminology or unclear results, the corresponding authors were contacted by e-mail to request additional information. As no responses were obtained, the information was reported in the data extraction table as it was stated in each study, alongside an explanation of why the relevant point was unclear. The senior author reviewed 5% of the retrieved articles (*n* = 91) as a check on the shortlisting process, and reviewed 5% of the shortlisted articles (*n* = 2) as a check on the data extraction process.

### Strength of association

Some quantitative studies reported associations between salmonellae infections and broader societal impacts, i.e. a very general relationship describing how one variable provides information about another. Other quantitative studies provided a more specific measure of the strength of relationship between the two variables. When associations between salmonellae infections and broader societal impacts were expressed through odds ratios (ORs) or risks ratios (RR) of the odds/risk of broader societal impacts for salmonellae infections compared to the odds/risk of broader societal impacts in a control group, we categorised the strength of association as very strong (OR > 4.0), strong (>2.5-4.0), moderate (>1.5–2.5), or weak (1.0–1.5). When associations between salmonellae infections and broader societal impacts were expressed through correlation coefficients, the strength of correlation was categorised using the qualitative descriptors as very strong (r 0.80–1.00), strong (0.60–0.79), moderate (0.40–0.59), weak (0.20–0.39), or very weak (0.00–0.19). For quantitative studies not reporting strengths of association due to the absence of a control group or of statistical analyses, and for all qualitative studies, we recorded any observations relevant to the strength of observation (e.g. magnitude, qualitative indicators) in the data extraction form. For example, with an ecological study that reported trends over time for salmonellae infections and poverty without any measures of association, we simply reported the length of time over which the study was conducted. When heterogeneity of results was observed among studies exploring associations, causes of heterogeneity were extracted and recorded (e.g. differences in study settings, populations, the definitions or measurements of exposures and/or outcomes, identification of confounders, missing data and its management, and/or study quality).

### Risk of bias

Risk of bias was assessed using the Risk Of Bias In Non-randomised Studies - of Exposure (ROBINS-E) tool^34^ according to the following domains: bias due to confounding, bias arising from measurement of the exposure, bias in selection of participants into the study (or into the analysis), bias due to post-exposure interventions, bias due to missing data, bias arising from measurement of the outcome, bias in selection of the reported result, and overall risk of bias rating.

### Quality assessment

Articles were quality assessed using three different quality appraisal checklists relevant to the study design (Supplementary Tables 4–6). Non-economic quantitative studies reporting correlations and associations and qualitative studies were assessed using the respective quantitative^35^ and qualitative^36^ appraisal checklists from the National Institute for Health and Care Excellence (NICE). Cost-of-illness case-series were assessed using a quality appraisal checklist from the Institute of Health Economics^37^. When a study reported both quantitative and qualitative data, both appropriate quality appraisal checklists were used and both results reported. Based on these three checklists, study quality was assessed to be low, good, or very good. As the studies included in the review were heterogeneous in the outcomes being assessed, a single framework for summarising the evidence could not be used.

### Conceptual framework

**Fig. 2:**
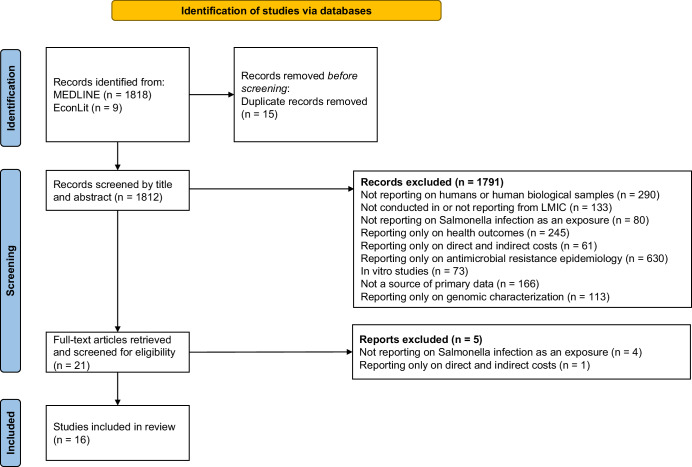
PRISMA flow diagram of database search and study selection^38^. The figure shows how the 16 studies included in the review were selected.

A conceptual framework of pathways between salmonellae infections and their proposed broader societal impacts was developed (Fig. 2, Table 1). This framework builds on a previously published generic framework for vaccine benefits^7^. We reframed this in terms of the impact of *Salmonella* disease itself (as we found no studies looking at the broader societal impacts of *Salmonella* vaccines), to focus on socio-economic outcomes, and to focus on the broader societal impacts identified in studies included in this review (e.g. childhood development, food insecurity).

## Supplementary information

Supplementary information

## Data Availability

All extracted data are presented in the Supplementary Information.
