# Supplementary material for: A systematised review and evidence synthesis on the broader societal impact of vaccines against Salmonella
Source: NPJ Vaccines. 2025 Feb 1;10:21. doi: 10.1038/s41541-024-01034-4 (PMC11787315; doi:10.1038/s41541-024-01034-4)
Supplement: Supplementary file 1 — Supplementary information [file 41541_2024_1034_MOESM1_ESM.pdf]

Supplementary Material to "A systematized review and evidence synthesis on the broader societal impact of vaccines against *Salmonella*."

Table of contents

1. Supplementary Table 1. PRISMA checklist. 1
2. Search strategy 5
   2.1. Supplementary Table 2. Summary of MEDLINE search strategy. 5
   2.2. Supplementary Table 3. Summary of EconLit search strategy. 7
3. Quality of studies. 10
   3.1. Note 1. Abbreviations used. 10
   3.2. Supplementary Table 4. Quality appraisal checklist for quantitative studies reporting correlations and associations. 11
   3.3. Supplementary Table 5. Quality appraisal checklist for qualitative studies. 12
   3.4. Supplementary Table 6. Quality appraisal checklist for case series. 13
4. Supplementary Table 7. Summary of studies. 15
5. Supplementary Table 8. Time points at which infections and childhood impacts were measured for studies with childhood physical development impacts (C1 in the framework). 17
6. Supplementary Table 9. Strength of association in studies reporting statistical measures 18
7. Supplementary references 19

1. Supplementary Table 1. PRISMA checklist.

Section and Topic	Item #	Checklist item	Location where item is reported
**TITLE**			
Title	1	Identify the report as a systematic review.	p.1
**ABSTRACT**			
Abstract	2	See the PRISMA 2020 for Abstracts checklist.	p.2
**INTRODUCTION**			
Rationale	3	Describe the rationale for the review in the context of existing knowledge.	p.3
Objectives	4	Provide an explicit statement of the objective(s) or question(s) the review addresses.	p.4
**METHODS**			
Eligibility criteria	5	Specify the inclusion and exclusion criteria for the review and how studies were grouped for	p.4

Section and Topic	Item #	Checklist item	Location where item is reported
		the syntheses.	
Information sources	6	Specify all databases, registers, websites, organisations, reference lists and other sources searched or consulted to identify studies. Specify the date when each source was last searched or consulted.	p.4
Search strategy	7	Present the full search strategies for all databases, registers and websites, including any filters and limits used.	Appendix p.5-10
Selection process	8	Specify the methods used to decide whether a study met the inclusion criteria of the review, including how many reviewers screened each record and each report retrieved, whether they worked independently, and if applicable, details of automation tools used in the process.	p.10
Data collection process	9	Specify the methods used to collect data from reports, including how many reviewers collected data from each report, whether they worked independently, any processes for obtaining or confirming data from study investigators, and if applicable, details of automation tools used in the process.	p.4,10
Data items	10a	List and define all outcomes for which data were sought. Specify whether all results that were compatible with each outcome domain in each study were sought (e.g. for all measures, time points, analyses), and if not, the methods used to decide which results to collect.	p.15
	10b	List and define all other variables for which data were sought (e.g. participant and intervention characteristics, funding sources). Describe any assumptions made about any missing or unclear information.	p.4
Study risk of bias assessment	11	Specify the methods used to assess risk of bias in the included studies, including details of the tool(s) used, how many reviewers assessed each study and whether they worked independently, and if applicable, details of automation tools used in the process.	p.5,10
Effect measures	12	Specify for each outcome the effect measure(s) (e.g. risk ratio, mean difference) used in the	p.4,5

Section and Topic	Item #	Checklist item	Location where item is reported
		synthesis or presentation of results.	
Synthesis methods	13a	Describe the processes used to decide which studies were eligible for each synthesis (e.g. tabulating the study intervention characteristics and comparing against the planned groups for each synthesis (item #5)).	p.4,5
	13b	Describe any methods required to prepare the data for presentation or synthesis, such as handling of missing summary statistics, or data conversions.	p.4,5
	13c	Describe any methods used to tabulate or visually display results of individual studies and syntheses.	p.13
	13d	Describe any methods used to synthesize results and provide a rationale for the choice(s). If meta-analysis was performed, describe the model(s), method(s) to identify the presence and extent of statistical heterogeneity, and software package(s) used.	p.5
	13e	Describe any methods used to explore possible causes of heterogeneity among study results (e.g. subgroup analysis, meta-regression).	p.5
	13f	Describe any sensitivity analyses conducted to assess robustness of the synthesized results.	None
Reporting bias assessment	14	Describe any methods used to assess risk of bias due to missing results in a synthesis (arising from reporting biases).	p.5
Certainty assessment	15	Describe any methods used to assess certainty (or confidence) in the body of evidence for an outcome.	p.5
**RESULTS**			
Study selection	16a	Describe the results of the search and selection process, from the number of records identified in the search to the number of studies included in the review, ideally using a flow diagram.	p.6,18
	16b	Cite studies that might appear to meet the inclusion criteria, but which were excluded, and explain why they were excluded.	p.18
Study characteristics	17	Cite each included study and present its characteristics.	p.6

Section and Topic	Item #	Checklist item	Location where item is reported
Risk of bias in studies	18	Present assessments of risk of bias for each included study.	p.16
Results of individual studies	19	For all outcomes, present, for each study: (a) summary statistics for each group (where appropriate) and (b) an effect estimate and its precision (e.g. confidence/credible interval), ideally using structured tables or plots.	Appendix p.15-17
Results of syntheses	20a	For each synthesis, briefly summarise the characteristics and risk of bias among contributing studies.	p.6-9
	20b	Present results of all statistical syntheses conducted. If meta-analysis was done, present for each the summary estimate and its precision (e.g. confidence/credible interval) and measures of statistical heterogeneity. If comparing groups, describe the direction of the effect.	None
	20c	Present results of all investigations of possible causes of heterogeneity among study results.	p.6-9
	20d	Present results of all sensitivity analyses conducted to assess the robustness of the synthesized results.	None
Reporting biases	21	Present assessments of risk of bias due to missing results (arising from reporting biases) for each synthesis assessed.	p.16
Certainty of evidence	22	Present assessments of certainty (or confidence) in the body of evidence for each outcome assessed.	p.6-9
**DISCUSSION**			
Discussion	23a	Provide a general interpretation of the results in the context of other evidence.	p.9
	23b	Discuss any limitations of the evidence included in the review.	p.9,10
	23c	Discuss any limitations of the review processes used.	p.10
	23d	Discuss implications of the results for practice, policy, and future research.	p.10
**OTHER INFORMATION**			
Registration and	24a	Provide registration information for the review, including register name and registration	None

Section and Topic	Item #	Checklist item	Location where item is reported
protocol		number, or state that the review was not registered.	
	24b	Indicate where the review protocol can be accessed, or state that a protocol was not prepared.	None
	24c	Describe and explain any amendments to information provided at registration or in the protocol.	None
Support	25	Describe sources of financial or non-financial support for the review, and the role of the funders or sponsors in the review.	p.11
Competing interests	26	Declare any competing interests of review authors.	p.11
Availability of data, code and other materials	27	Report which of the following are publicly available and where they can be found: template data collection forms; data extracted from included studies; data used for all analyses; analytic code; any other materials used in the review.	Data extracted: Appendix p.10-17

2. Search strategy

2.1. Supplementary Table 2. Summary of MEDLINE search strategy.

Search line	Search terms: keywords and synonyms	Number of hits	Search line	Search terms: subject headings	Number of hits
Disease					
1	(salmonella or typhoid or paratyphoid or iNTS).mp.	112,870	2	exp Salmonella typhi/ or exp Salmonella paratyphi B/ or exp Salmonella typhimurium/ or exp Salmonella enteritidis/ or exp Salmonella paratyphi C/ or exp Salmonella paratyphi A/ or exp Salmonella Infections/ or exp	84,954

				Salmonella enterica/ or exp Salmonella/ or exp Salmonella Vaccines/ or Salmonella arizonae/ or exp paratyphoid fever/ or exp typhoid fever/	
Burden					
3	(societ* impact or cost* or financ*).mp.	1,918,293	4	Community resources/ or social integration/ or exp cost-benefit analysis/ or exp cost-effectiveness analysis/ or "cost control"/ or "cost savings"/ or "cost of illness"/ or "global burden of disease"/ or health care costs/	180,481
	(antibiotic resistance or antimicrobial resistance).mp.		5	exp beta-lactam resistance/ or exp drug resistance, multiple, bacterial/ or exp trimethoprim resistance/	51,494
	(child* develop*).mp.		6	exp Child Development/	66,149
	(education* outcome* or access AJD1 education*).mp.		7	exp educational status/ or exp academic failure/ or exp literacy/	59,689
	(fertility).mp.		8	Fertility/	44,058
	(female labo?r).mp.		9	Women, Working/	5,580
	(equit* or equality or fairness or inequity or inequality or disparity).mp.		10	Health Equity/	3,373
	(poverty).mp.		11	exp Poverty/	49,501
	(consumption or saving or investment*		12	economic stability/ or housing instability/	94

	or house* financ* security).mp.				
	(macro-economic or tax or taxes or taxation or gdp or gross domestic product or nation* growth or nation* income or foreign investment*).mp.		13	exp economic development/ or gross domestic product/	6,870
	(house* behavio?r).mp.				
	(programm* synerg*).mp.				
	-		14	"Health Services Needs and Demand"/	55,115
Combined results					
8	1 or 2				112,870
9	3 or 4 or 5 or 6 or 7 or 8 or 9 or 10 or 11 or 12 or 13 or 14				2,070,905
10	Expert search "Medline all countries designated as LMIC"				1,847,973
11	8 and 9 and 10 (limits applied*)				1,813

*limited to records published in English or French, and studies involving human participants only

2.2. Supplementary Table 3. Summary of EconLit search strategy.

Search line	Search terms: keywords and synonyms	Number of hits	Search line	Search terms: subject headings	Number of hits
Disease					
1	(salmonella or typhoid or paratyphoid or iNTS).mp.	78		-	-
Burden					
2	(societ* impact or cost* or financ*).mp.	859,017	3	financial crises.sh.	23,506
	(antibiotic resistance or		4	-	-

	antimicrobial resistance).mp.				
	(child* develop*).mp.		5	-	-
	(education* outcome* or access AJD1 education*).mp.		6	("education and economic development" or "education and inequality" or "education and research institutions general" or education government policy or education other or educational finance financial aid).sh.	23,902
	(fertility).mp.		7	fertility family planning child care children youth.sh.	28,203
	(female labo?r).mp.		8	-	-
	(equit* or equality or fairness or inequity or inequality or disparity).mp.		9	(equities fixed income securities or "equity justice inequality and other normative criteria and measurement").sh.	32,992
	(poverty).mp.		10	-	-
	(consumption or saving or investment* or house* financ* security).mp.		11	(household saving borrowing debt and wealth or household saving personal finance).sh.	4,305
	(macro-economic or tax or taxes or taxation or gdp or gross domestic product or nation* growth or nation* income or foreign investment*).mp.		12	(“macroeconomic analyses of economic development” or "macroeconomic aspects of international trade and finance forecasting and simulation models and applications" or "macroeconomic aspects of international trade and finance general" or "macroeconomic aspects of international trade and finance other" or "macroeconomic policy macroeconomic aspects of public finance and general outlook general" or "macroeconomic policy	69,693

				macroeconomic aspects of public finance and general outlook other" or "macroeconomics and monetary economics general" or "macroeconomics consumption saving production employment and investment forecasting and simulation models and applications" or macroeconomics consumption saving wealth or macroeconomics production).sh.	
				("taxation and other revenue general" or "taxation and other revenue history" or "taxation and other revenue state and local").sh.	522
				("growth and development theory factor proportions" or "growth and development theory general" or "growth and development theory underdeveloped economies" or growth models or growth stagnation in developed economies or growth theories general).sh.	1965
				(national income accounts or "national wealth and balance sheets").sh.	1640
				("international investment and long term capital movements" or "international investment and long term capital movements general" or "international investment and long term capital movements studies"	18007

				or "international investment and long term capital movements theory" or international investment long term capital movements).sh.	
	(house* behavio?r).mp.		13	("household behavior and family economics other" or household behavior general or household finance financial literacy or household finance general or household finance insurance or household finance other or "household production and intrahousehold allocation").sh.	18483
	(programm* synerg*).mp.		14	-	
Combined results					
8	2 or 3 or 4 or 5 or 6 or 7 or 8 or 9 or 10 or 11 or 12				888173
9[a]	1 and 13 (limits applied)				42

[a]Limited to records published in English and French

3. Quality of studies.

3.1. Note 1. Abbreviations used.

Study design

C	cross-sectional
E	ecological
P	prospective case-control

Quality level for individual items

++	Indicates that for that particular aspect of study design the study has been designed or conducted in such a way as to minimise the risk of bias.
+	Indicates that either the answer to the checklist question is not clear from the way the study is reported, or that the study may not have addressed all potential sources of bias for that particular aspect of study design

-	Should be reserved for those aspects of the study design in which significant sources of bias may persist.
NR	Not reported: Should be reserved for those aspects in which the study under review fails to report how they have (or might have) been considered.
NA	Not applicable: Should be reserved for those study design aspects that are not applicable given the study design under review

Quality level for overall study quality appraisal

++	All or most of the checklist criteria have been fulfilled, where they have not been fulfilled the conclusions are very unlikely to alter.
+	Some of the checklist criteria have been fulfilled, where they have not been fulfilled, or not adequately described, the conclusions are unlikely to alter.
-	Few or no checklist criteria have been fulfilled and the conclusions are likely or very likely to alter.

3.2. Supplementary Table 4. Quality appraisal checklist for quantitative studies reporting correlations and associations.

Reference	[1]	[2]	[3]	[4]	[5]	[6]	[7]	[8]	[9]	[10]	[11]
Design	E	E	E	E	P	E	C	C	C	E	E
Section 1: Population											
1.1 Is the source population or source area well described?	++	+	+	++	++	++	++	++	++	+	++
1.2 Is the eligible population or area representative of the source population or area?	-	-	-	-	-	-	-	-	-	-	-
1.3 Do the selected participants or areas represent the eligible	++	++	++	++	+	++	++	++	++	-	++
Section 2: Method of selection of exposure (or comparison) group											
2.1 Selection of exposure (and comparison) group. How was selection bias minimised?	-	-	-	-	++	-	NR	++	++	-	-
2.2 Was the selection of explanatory variables based on a sound theoretical basis?	++	++	++	++	++	++	++	++	++	++	++
2.3 Was the contamination acceptably low?	-	-	-	-	+	-	NR	+	+	-	-

2.4 How well were likely confounding factors identified and controlled?	-	-	-	-	+	-	-	-	+	-	-
Section 3: Outcomes											
3.1 Were the outcome measures and procedures reliable?	++	+	++	+	++	++	NR	+	+	+	+
3.2 Were the outcome measurements complete?	+	++	++	++	++	NR	++	NR	++	NR	+
3.3 Were all the important outcomes assessed?	++	++	++	-	++	++	-	++	++	++	++
3.4 Was there a similar follow-up time in exposure and comparison groups?	++	++	++	++	++	++	NA	-	-	++	++
3.5 Was follow-up time meaningful?	+	+	+	++	++	++	NA	-	-	++	++
Section 4: Analyses											
4.1 Was the study sufficiently powered to detect an intervention effect (if one exists)?	-	-	+	+	++	++	NR	-	++	++	++
4.2 Were multiple explanatory variables considered in the analyses?	++	++	++	-	+	-	++	++	++	-	-
4.3 Were the analytical methods appropriate?	-	-	-	-	++	-	-	-	+	-	-
4.4 Was the precision of association given or calculable? Is association meaningful?	-	-	-	++	++	-	-	++	++	-	++
Section 5: Summary											
5.1 Are the study results internally valid (i.e. unbiased)?	-	-	-	-	++	-	-	-	+	-	-
5.2 Are the findings generalisable to the source population (i.e. externally valid)?	-	-	-	-	-	-	-	-	-	-	-
Overall study quality	-	-	-	-	++	-	-	-	+	-	-

3.3. Supplementary Table 5. Quality appraisal checklist for qualitative studies.

Reference	[12]
Theoretical approach	
1. Is a qualitative approach appropriate?	Appropriate
2. Is the study clear in what it seeks to do?	Clear

Study design	
3. How defensible / rigorous is the research design / methodology?	Defensible
Data collection	
4. How well was the data collection carried out?	Appropriately
Trustworthiness	
5. Is the role of the researcher clearly described?	Not described
6. Is the context clearly described?	Clear
7. Were the methods reliable?	Reliable
Analysis	
8. Is the data analysis sufficiently rigorous?	Rigorous
9. Is the data 'rich'?	Not sure / not reported
10. Is the analysis reliable?	Not sure / not reported
11. Are the findings convincing?	Convincing
12. Are the findings relevant to the aims of the study?	Relevant
Conclusions	
13. Is there adequate discussion of any limitations encountered?	Adequate
Ethics	
14. How clear and coherent is the reporting of ethics?	Appropriate
Overall assessment	
As far as can be ascertained from the paper, how well was the study conducted?	+

3.4. Supplementary Table 6. Quality appraisal checklist for case series.

Reference	[12]	[13]	[14]	[15]	[16]
Study objective					
1. Was the hypothesis/aim/objective of the study clearly defined?	Yes	Yes	Yes	Yes	Yes
Study design					
2. Was the study conducted prospectively?	No	No	Yes	Yes	No
3. Were the cases collected in more than one centre?	Yes	Yes	Yes	Yes	No
4. Were patients recruited consecutively?	Unclear	Yes	Unclear	Yes	No
Study population					
5. Were the characteristics of the patients included in the study described?	Partial	Yes	Yes	Partial	Yes
6. Were the eligibility criteria (i.e. inclusion and exclusion criteria) for entry	Yes	Yes	Yes	Partial	Yes

into the study clearly stated?					
7. Did patients enter the study at a similar point in the disease?	Unclear	No	Unclear	No	Yes
Intervention and co-intervention					
8. Was the intervention of interest clearly described?	Yes	Yes	Yes	Yes	Yes
9. Were additional interventions (co-interventions) clearly described?	Not applicable	Yes	Yes	Not applicable	Not applicable
Outcome measure					
10. Were relevant outcome measures established a priori?	Yes	Yes	Yes	Yes	Yes
11. Were outcome assessors blinded to the intervention that patients received?	No	No	No	No	No
12. Were the relevant outcomes measured using appropriate objective/subjective methods?	Yes	Yes	Yes	Yes	Partial
13. Were the relevant outcome measures made before and after the intervention?	Yes	Yes	Yes	Yes	Yes
Statistical analysis					
14. Were the statistical tests used to assess the relevant oucomes appropriate?	No	Yes	Unclear	Unclear	No
Results and conclusions					
15. Was follow-up long enough for important events and outcomes to occur?	Unclear	Unclear	Yes	Yes	Yes
16. Were losses to follow-up reported?	Yes	Yes	Yes	No	Not applicable
17. Did the study provide estimates of random variability in the data analysis of relevant outcomes?	Yes	Yes	Yes	Partial	No
18. Were the adverse events reported?	Not applicable	Not applicable	No	Not applicable	Yes

19. Were the conclusions of the study supported by results?	Yes	Yes	Unclear	Yes	Yes
Competing interests and sources of support					
20. Were both competing interests and sources of support for the study reported?	Yes	Yes	Yes	Partial	Yes
Overall study quality	-	+	+	-	-

4. Supplementary Table 7. Summary of studies.

Impact	Relevant studies	Strength of association	Study quality
B1 and B2. Productivity-related impacts	Kaljee, 2018 [12]	Not applicable (no control group)	+ (qualitative study)<br>- (case series)
B3. Childhood development (physical)	Das, 2021 [5]	After adjustment for numerous confounders, NTS was very weakly associated with decreasing weight-for-age and weight-for-height (correlation coefficients -0.19 for both)	++ (case-control study)
B3. Childhood development (educational)	Akinyemi, 2018 [1]	No association was explored (no statistical analyses)	- (ecological study)
	Balaji, 2018 [2]	No association was explored (no statistical analyses)	- (ecological study)
	Bhutta, 2018 [3]	No association was explored (no statistical analyses)	- (ecological study)
	Das, 2018 [4]	Adult female literacy strongly negatively associated with typhoid incidence (r=-0.81).<br><br>Adult literacy strongly negatively associated with typhoid incidence (r=-0.82).	- (ecological study)
	Kaljee, 2018 [12]	Not applicable (no control group)	Qualitative study did not discuss this area.<br><br>- (case series)
	Keddy, 2018 [6]	No association was explored (no statistical analyses)	- (ecological study)

	Poulos, 2011 [15]	Not applicable (no control group)	- (case series)
	Saha, 2018 [10]	No association was explored (no statistical analyses)	- (ecological study)
	Techasaensiri, 2018 [11]	No association was explored (no statistical analyses)	- (ecological study)
C2. Household financial security (distress financing)	Kaljee, 2018 [12]	Not applicable (no control group)	Qualitative study did not discuss this area.<br><br>- (case series)
	Kumar, 2021 [13]	Not applicable (no control group)	+ (case series)
	Rahman, 2013 [8]	Distress financing moderately positively associated with typhoid incidence (RR 1.92, 95% CI 1.08 to 3.43).	- (cross-sectional survey)
Household financial security (catastrophic health expenditure)	Limani, 2022 [14]	Not applicable (no control group)	- (case series)
	Poulos, 2011 [15]	Not applicable (no control group)	- (case series)
	Rahman, 2020 [9]	Not applicable (no control group)	+ (cross-sectional survey)
	Seyi-Olajide, 2020 [16]	Not applicable (no control group)	- (case series)
Household financial security (extreme poverty)	Akinyemi, 2018 [1]	No association was explored (no statistical analyses)	- (ecological study)
	Bhutta, 2018 [3]	No association was explored (no statistical analyses)	- (ecological study)
	Das, 2018 [4]	Poverty was strongly positively associated with typhoid incidence (correlation coefficient 0.79).	- (ecological study)
	Keddy, 2018 [6]	No association was explored (no statistical analyses)	- (ecological study)
	Saha, 2018 [10]	No association was explored (no statistical analyses)	- (ecological study)
	Techasaensiri, 2018 [11]	No evidence of association between poverty and either typhoid (regression coefficient 2.01, 95% CI -0.43 to 4.45, p=0.09)	- (ecological study)

		or paratyphoid (regression coefficient -0.03, 95% CI -0.38 to 0.31, p=08.2) incidence.	
Household food insecurity	Onuche, 2013[7]	No association was explored (no statistical analyses)	- (cross-sectional survey)
D2. Public sector budget impacts (public health spending)	Balaji, 2018 [2]	No association was explored (no statistical analyses)	- (ecological study)
	Das, 2018 [4]	Health expenditure moderately negatively associated with typhoid incidence (r=-0.45).<br><br>No evidence of association between health expenditure and paratyphoid incidence.	- (ecological study)
D3 and D4. Macro-economic impact	Akinyemi, 2018 [1]	No association was explored (no statistical analyses)	- (ecological study)
	Balaji, 2018 [2]	No association was explored (no statistical analyses)	- (ecological study)
	Keddy, 2018 [6]	No association was explored (no statistical analyses)	- (ecological study)
	Das, 2018 [4]	GNI per capita strongly negatively associated with typhoid incidence (r=-0.68).<br><br>GNI per capita moderately negatively associated with paratyphoid incidence (r=-0.49).	- (ecological study)

5. **Supplementary Table 8. Time points at which infections and childhood impacts were measured for studies with childhood physical development impacts (C1 in the framework).**

Study	Time period at which infection was recorded	Time period at which childhood development was recorded
Das, 2021 [5]	At enrolment	At enrolment and at 60 days follow-up (acceptable range 50-90 days)
Akinyemi, 2018 [1]	1993 to 2017	2008 to 2015
Balaji, 2018 [2]	2000 to 2015	1991 to 2015
Bhutta, 2018 [3]	2000 to 2016	1990 to 2015
Das, 2018 [4]	1990 to 2015	1990 to 2015

Kaljee, 2018 [12]	Within the past 6 months	During cross-sectional surveys following an infection in the last 6 months
Keddy, 2018 [6]	1980 to 2015	1980 to 2012
Poulos, 2011 [15]	2001 to 2004	Survey at 7, 14, and 90 days after onset of illness
Saha, 2018 [10]	2001 to 2014	1990 to 2014
Techasaensiri, 2018 [11]	2003 to 2014	2003 to 2014

6. Supplementary Table 9. Strength of association in studies reporting statistical measures

Impact	Study	Strength of association
A3. Public sector budget impacts (public health spending)	Das, 2018 [4]	The increasing exposure health expenditure was moderately associated with the decreasing outcome typhoid incidence (correlation coefficient -0.45, p-value 0.05) There was no evidence of association between the exposure health expenditure and the outcome paratyphoid incidences (correlation coefficient 0.09, p-value 0.70)
A4. Macro-economic impacts (GNI)	Das, 2018 [4]	The increasing exposure GNI per capita was strongly associated with the decreasing outcome typhoid incidence (correlation coefficient -0.68, p-value<0.001) The increasing exposure GNI per capita was moderately associated with the decreasing outcome paratyphoid incidence (correlation coefficient -0.49, p-value 0.01)
C1. Childhood physical development<br><br>Childhood educational development	Das, 2021 [5]<br><br>Das, 2018 [4]	After adjustment for numerous confounders, NTS was very weakely associated with decreasing weight-for-age and weight-for-height (for weight-for-age correlation coefficient -0.19, 95%CI -0.33 to -0.04, p-value 0.01; for weight-for-height correlation coefficient -0.19, 95%CI -0.34 to -0.05, p-value 0.007). There was no evidence of association between NTS and height-for-age (correlation coefficient -0.13, 95%CI -0.27 to -0.01, p-value 0.07)<br>The increasing exposure adult female literacy was very strongly associated with the decreasing outcome typhoid incidence (correlation coefficient -0.81, p-value 0.005) The increasing exposure adult literacy was very strongly associated with the decreasing

		outcome typhoid incidence (correlation coefficient -0.82, p-value 0.003)
C2. Household financial security (distress financing)<br>Household financial security (poverty)	Rahman, 2013 [8]<br><br>Das, 2018 [4]<br><br><br>Techasaensiri, 2018 [11]	Typhoid was moderately associated (RR 1.92, 95%CI 1.08 to 3.43) with distress financing<br><br>The decreasing exposure poverty was strongly associated with the decreasing outcome typhoid incidence (correlation coeffiient 0.79, p-value 0.02)<br>There was no evidence of association between the exposure poverty and the outcomes typhoid and paratyphoid incidences:<br>- S.Typhi: regression coefficient 2.01 (95%CI -0.43 to 4.45, p-value 0.09)<br>- S.Paratyphi: regression coefficient -0.03 (95%CI -0.38 to 0.31, p-value 0.82)

7. Supplementary references

[1] Akinyemi KO, Oyefolu AOB, Mutiu WB, Iwalokun BA, Ayeni ES, Ajose SO, et al. Typhoid Fever: Tracking the Trend in Nigeria. Am J Trop Med Hyg 2018;99:41–7. https://doi.org/10.4269/ajtmh.18-0045.

[2] Balaji V, Kapil A, Shastri J, Pragasam AK, Gole G, Choudhari S, et al. Longitudinal Typhoid Fever Trends in India from 2000 to 2015. Am J Trop Med Hyg 2018;99:34–40. https://doi.org/10.4269/ajtmh.18-0139.

[3] Bhutta ZA, Gaffey MF, Crump JA, Steele D, Breiman RF, Mintz ED, et al. Typhoid Fever: Way Forward. Am J Trop Med Hyg 2018;99:89–96. https://doi.org/10.4269/ajtmh.18-0111.

[4] Das JK, Hasan R, Zafar A, Ahmed I, Ikram A, Nizamuddin S, et al. Trends, Associations, and Antimicrobial Resistance of Salmonella Typhi and Paratyphi in Pakistan. The American Journal of Tropical Medicine and Hygiene 2018;99:48–54. https://doi.org/10.4269/ajtmh.18-0145.

[5] Das R, Haque MA, Chisti MJ, Faruque ASG, Ahmed T. Association between Non-Typhoidal Salmonella Infection and Growth in Children under 5 Years of Age: Analyzing Data from the Global Enteric Multicenter Study. Nutrients 2021;13:392. https://doi.org/10.3390/nu13020392.

[6] Keddy KH, Smith AM, Sooka A, Tau NP, Ngomane HMP, Radhakrishnan A, et al. The Burden of Typhoid Fever in South Africa: The Potential Impact of Selected Interventions. Am J Trop Med Hyg 2018;99:55–63. https://doi.org/10.4269/ajtmh.18-0182.

[7] Onuche U, Edoka M. Effect of Health Status and Selected Household Characteristics on Food Security among Rural Households of Kogi State, Nigeria. International Journal of Applied Economics and Econometrics 2013;21:83–96.

[8] Rahman MM, Gilmour S, Saito E, Sultana P, Shibuya K. Self-reported illness and household strategies for coping with health-care payments in Bangladesh. Bull World Health Organ 2013;91:449–58. https://doi.org/10.2471/BLT.12.115428.
[9] Rahman MM, Zhang C, Swe KT, Rahman MS, Islam MR, Kamrujjaman M, et al. Disease-specific out-of-pocket healthcare expenditure in urban Bangladesh: A Bayesian analysis. PLOS ONE 2020;15:e0227565. https://doi.org/10.1371/journal.pone.0227565.
[10] Saha S, Saha S, Das RC, Faruque ASG, Salam MA, Islam M, et al. Enteric Fever and Related Contextual Factors in Bangladesh. Am J Trop Med Hyg 2018;99:20–5. https://doi.org/10.4269/ajtmh.18-0106.
[11] Techasaensiri C, Radhakrishnan A, Als D, Thisyakorn U. Typhoidal Salmonella Trends in Thailand. Am J Trop Med Hyg 2018;99:64–71. https://doi.org/10.4269/ajtmh.18-0046.
[12] Kaljee LM, Pach A, Garrett D, Bajracharya D, Karki K, Khan I. Social and Economic Burden Associated With Typhoid Fever in Kathmandu and Surrounding Areas: A Qualitative Study. The Journal of Infectious Diseases 2018;218:S243–9. https://doi.org/10.1093/infdis/jix122.
[13] Kumar D, Sharma A, Rana SK, Prinja S, Ramanujam K, Karthikeyan AS, et al. Cost of Illness Due to Severe Enteric Fever in India. J Infect Dis 2021;224:S540–7. https://doi.org/10.1093/infdis/jiab282.
[14] Limani F, Smith C, Wachepa R, Chafuwa H, Meiring J, Noah P, et al. Estimating the economic burden of typhoid in children and adults in Blantyre, Malawi: A costing cohort study. PLOS ONE 2022;17:e0277419. https://doi.org/10.1371/journal.pone.0277419.
[15] Poulos C, Riewpaiboon A, Stewart JF, Clemens J, Guh S, Agtini M, et al. Cost of illness due to typhoid fever in five Asian countries. Trop Med Int Health 2011;16:314–23. https://doi.org/10.1111/j.1365-3156.2010.02711.x.
[16] Seyi-Olajide JO, Anderson J, Enivwaene AO, Ibrahim SH, Farmer D, Ameh EA. Catastrophic Healthcare Expenditure from Typhoid Perforation in Children in Nigeria. Surg Infect (Larchmt) 2020;21:586–91. https://doi.org/10.1089/sur.2020.134.
